# Opium Antidotes

**Published:** 1877-05-20

**Authors:** 


OPIUM ANTIDOTES.—Dr. J. B. Mattison, of New York, read before the Medical Society of the county of Kings, Brooklyn, an interesting paper exposing a reputed antidote for the opium habit, gotten up by one S. B. Collins, of Indiana, and which is having an extensive sale. He had it analyzed with the following result.

| | |
|---|---|
| Water | 28.66 |
| Glycerine | 66.88 |
| *Sulphate of Morphia* | 4.45 |

The sample is colored with magenta.

Analysis by Walz and Stillwell.

This proportion of morphine amounts to more than *grains* xxv. *to the ounce!*

An analysis of the same nostrum, made by another party, confirms this report; and the same is true of the so-called antidote of Mrs. J. A. Drollinger.

